# Modeling record scores in the snatch and its variations in the long-term training of young weightlifters

**DOI:** 10.1371/journal.pone.0225891

**Published:** 2019-12-03

**Authors:** Adam Czaplicki, Paulina Szyszka, Jarosław Sacharuk, Janusz Jaszczuk

**Affiliations:** 1 Department of Natural Sciences, Faculty of Physical Education and Health, Jozef Pilsudski University of Physical Education, Warsaw, Poland; 2 Department of Sport Sciences, Faculty of Physical Education and Health, Jozef Pilsudski University of Physical Education, Warsaw, Poland; São Paulo State University (UNESP), BRAZIL

## Abstract

The primary aim of the current study was to determine the time curves of changes in the record scores in the snatch and its variations during a two-year training cycle in young weightlifters. This study also aimed at assessing the ratios between these scores and at predicting the snatch record scores at the end of the subsequent annual training macrocycle. The final purpose was to compare the record scores with the isometric peak torque values of the trunk and knee extensors. The study involved 16 weightlifters who were tested seven times at three-month intervals. The overall mean ratios of the record scores in the hang snatch to those in the snatch and the record scores in the hang power snatch to those in the snatch were approximately constant and amounted to 0.95 and 0.79, respectively. The overall mean ratio between the scores in the power snatch to those in the snatch was approximately 0.88. Statistically significant differences (*p* < 0.05) between the individual time trajectories of record scores in the snatch and its derivatives were identified in two consecutive annual training macrocycles. The error in predicting record results at the end of the following annual training macrocycle was 6.7 ± 4.7% or 8.1 ± 3.4% depending on the way the measurement data were modeled. The results of the study also indicate that the measurements of the isometric peak torque of the trunk extensors performed in laboratory conditions can be useful in diagnosing the strength capacity of young weightlifters.

## Introduction

Elite weightlifters typically perform 20,000–25,000 lifts per year. Most of the lifts are done with a load equal to 80–90% of maximal capacity, and 4–7% are executed at more than 90% of 1 repetition maximum (1 RM) [1,2]. Drechsler [3] reports that in weightlifting training, 10% of the total time is dedicated to warm-ups, 45% to competitions and specific exercise, 40% to complementary strength exercise, 3% to supplementary exercise, and 2% to other sport- and training-related activities. The heavy use of specific exercise in training programs indicates that it is expected to have a direct influence on improving weightlifters’ performance.

The use of weightlifting exercises has previously been shown to enhance strength, power, and speed, hence its popularity within performance training programs in different sport disciplines [4–12]. In a survey presented in the work of Waryasz et al. [13], 92% out of 167 crossfit trainers used weightlifting exercise in their training programs, and a study by Smith et al. [14] showed that 94.2% applied the snatch and/or its variations.

The basic variations of the snatch (S) are the power snatch (PS), the hang power snatch (HPS), and the hang snatch (HS). A characteristic feature of the power snatch is that the bar is only lifted for a short time, since the lifter does not drop into a full catch position [15–16]. Moreover, smaller loads are used in this exercise, thanks to which the barbell can attain higher vertical velocity. Taking into account the two above-mentioned aspects of the power snatch, it is presumed that incorporating this exercise in the training causes beneficial neuromuscular adaptations, which increase the power generated by the lifter’s muscles [7,9,17]. Power is regarded as a valuable predictor of weightlifters’ performance [18]. It can be defined as a product of the weight of the barbell and its vertical velocity (the power of the barbell) and can be used in assessing exercise intensity [15].

Hang snatches are variations of the snatch where the starting position of the barbell is above the lifter’s knees. This initial configuration of the body helps the lifter achieve triple extension, that is extension in the hip, knee, and ankle joints [11,17,19–20]. A smaller difference between the starting and finishing position of the barbell makes it easier to master the technique of performing the hang snatch and the hang power snatch. Owing to the fact that these exercises do not include the technically difficult elements that are characteristic of the snatch, they are recommended for athletes with shorter training experience, and their use increases the strength and power capacities of the muscles [15,20].

Snatch derivatives are used mainly as supplementary exercises aimed at improving the technique and outcomes of the snatch in weightlifters [2,21]. These exercises have also been found to be beneficial for enhancing motor capacity. Training incorporating the hang snatch was observed to produce improvements in vertical jump height, 1-RM back squats, and 40-yard sprint in female collegiate students [19]. A study by Canavan et al. [22] showed that this exercise was useful in developing the power of the lower extremities in football players and track field athletes. Other authors have noted a strong correlation between power snatch and shot put/weight throw results in well-trained collegiate throwers [23] as well as between the results in the hang snatch (and hang power snatch) and isometric knee extensor torque in young weightlifters [24].

Despite the extensive use of the snatch and its derivatives in training programs in different sport disciplines, there is no research available identifying time trends in the snatch record scores and its derivatives over several years of training. Neither has it been investigated whether there is a similarity between increases in lifters’ record scores in the snatch and increases in the peak torque of the extensors of the lower extremities responsible for the triple extension. One way to examine these issues is by long-term modeling of empirical data from measurements conducted at regular intervals.

In light of the above, the primary aim of the study was to determine the time curves of changes in the record scores in the snatch and its variations during a two-year training cycle in young weightlifters. This study also aimed at assessing the ratios between these scores and at predicting the snatch record scores at the end of the subsequent annual training macrocycle. The final purpose was to investigate the increases in the peak torque of the trunk and knee extensors and verify whether these increases corresponded with those in the record scores in the snatch in the period analyzed.

## Materials and methods

### Subjects

The study involved 16 weightlifters, who were selected from a training group of 25 persons. The criterion for participation in the research was at least one year of training experience and age below 20 years. One year of specific weightlifting training meant participation in 2-hour training sessions at least three times a week, during which the techniques of the snatch, the clean and jerk, and specific exercises were taught and improved. After a one-year training cycle, the weightlifter was able to correctly perform the basic lifts and exercises, including those whose performance was analyzed in this article. When the research began, the lifters were 16.5 ± 3.63 years old and had a body mass of 73.38 ± 18.91 kg. The participants included medalists in Polish championships in particular age categories and members of Polish national teams in European and World Championships. The study was carried out over a two-year period of specialized weightlifting training. The type of training and the percentage contribution of specific exercises to the training loads were similar to those described in our previous work [24]. During this period, the lifters prepared to participate in two main competitions (Youth European Weightlifting Championships in September 2016 and September 2017), and during the preparatory phases, they took part in regional and national tournaments. Eight subjects continued their training in the following year, which made it possible to conduct an additional measurement session at the end of the following training macrocycle in September 2018. Five of the eight weightlifters who did not continue their participation in the research changed their place of residence and club membership because they started their studies, while three withdrew for health or personal reasons. The subjects were provided with information on the research procedure and on the possible risks and benefits related to participating in the study. All subjects and their parents signed informed consent forms. The investigations were performed in accordance with the ethical standards of the Helsinki Declaration, and the study was approved by the University Research Ethics Committee.

### Measurements

The record scores in the snatch and its derivatives as well as isometric peak torque values were measured every three months in the first week of the months given in Table 1. All of these assessments were made on Mondays because Sunday was a day without training regardless of the phase of the macrocycle. That way the weightlifters were not tired before the measurement sessions. Over the following four days, the lifters’ maximal performance in the snatch and its three derivatives was tested. The tests were performed in a random order. There are 24 permutations of 4 measurement sessions; seven permutations were randomly selected by the computer without repetitions. Each test was preceded with a warm-up, during which loads no greater than 90% of the lifters’ existing personal best scores were used. Afterwards, the lifters performed 3 maximal lifts with progressive increases in load, starting from a load equal to or greater than 90% of 1 RM. The mean increase in the loads between consecutive repetitions was 2 or 3 kg. The repetition with the highest load (1 RM) was recorded as the final result of the test. Owing to the differences in body mass between subjects and its within-subject variations over the course of the study, the lifters’ body mass changes were recorded regularly (Table 1), and the results achieved in particular lifts were converted into Sinclair points. Sinclair coefficients were calculated according to the formula valid from 1 January 2017 to 31 December 2020.

**Table 1:** Mean body mass of weightlifters during the study in kg (± SD).

Measurement	M_1_	M_2_	M_3_	M_4_	M_5_	M_6_	M_7_
Date	March 2016	June 2016	September 2016	December 2016	March 2017	June 2017	September 2017
**Body mass** **(SD)**	73.38 (18.91)	74.28 (18.89)	75.75 (17.86)	76.51 (18.41)	77.34 (20.22)	78.91 (20.15)	79.41 (19.96)

**Fig 1:**
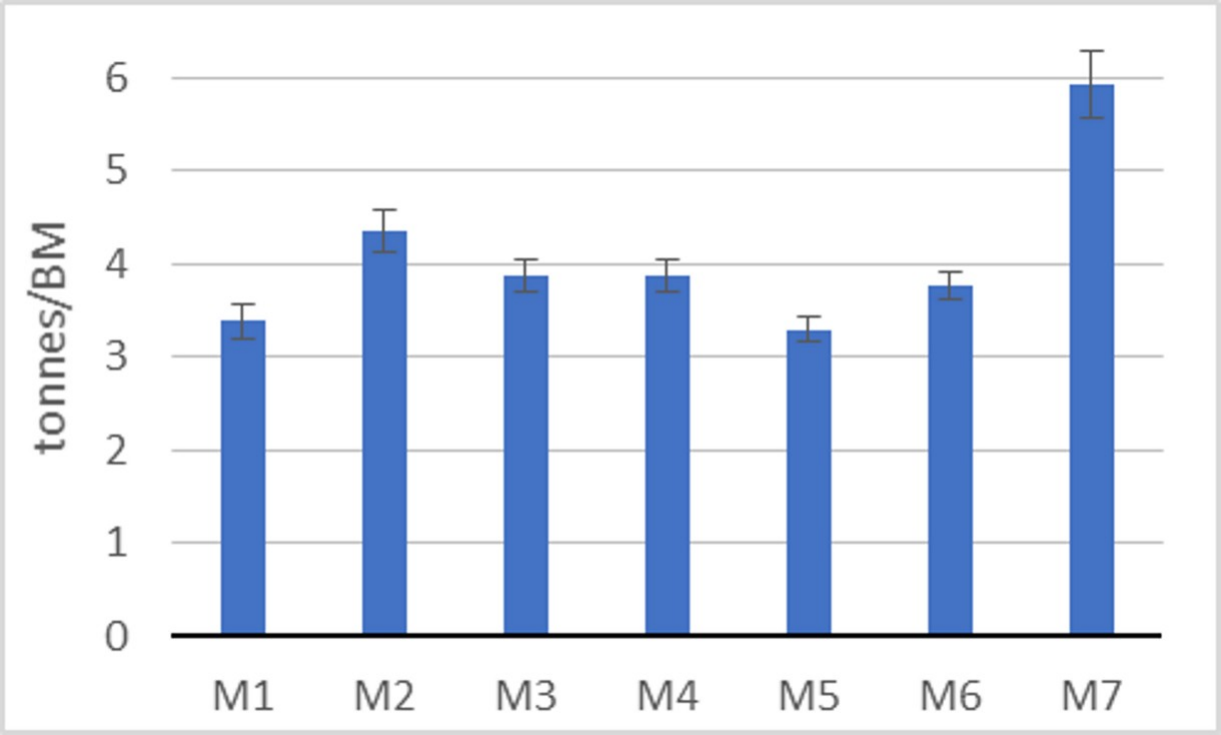
Relative training volume during the period analyzed.

During the study, the training loads that were implemented were also continuously monitored (Fig 1). The values of loads at measuring points *M*_*1*_-*M*_*7*_ are the sums of loads from the quarter preceding the measurement and were standardized in relation to the subjects’ body mass (BM).

**Fig 2:**
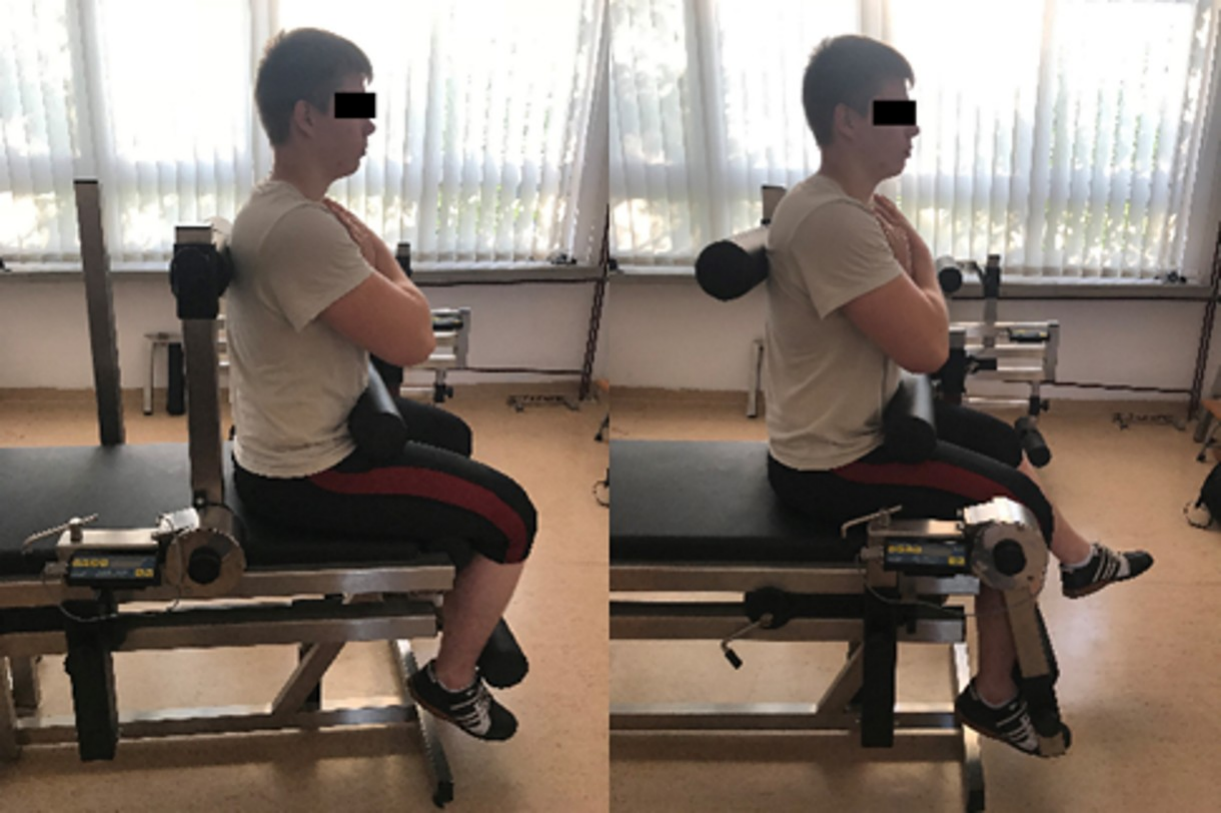
Measurement of peak torque of trunk extensors (left) and knee extensors (right) in isometric conditions.

The peak torque values of the muscles of the lower extremities and trunk were measured in isometric conditions [9,25–26]. Considering the decisive influence of the trunk and knee extensors on performance in weightlifting, only the torque of these two muscle groups was measured. The measurements were carried out on a LR2-P (JBA Zb. Staniak, Poland) measuring station [27]. The subjects adopted a standard position on a chair and were stabilized with back, thigh, and ankle pads, as shown in Fig 2. The angle in the hip and knee joints was 90 degrees, similarly as in the above mentioned papers. During the test, the lifters completed three repetitions of extension for no longer than 3 seconds. The current torque values were displayed and recorded by a dynamometer, and the best score was used in the analysis.

### Statistical analysis

The empirical data were tested for normality of distribution using the Shapiro-Wilk test. Statistical significance was set at *p* < 0.05. The results of the test confirmed that the data were normally distributed. The data were then represented in a univariate form. The time of the first measurement session was set to 0. Due to the fact that the measurements were carried out quarterly, the time of subsequent sessions increased by 0.25. By coding the first time period as 0, we directly attributed the intercept to the value of the dependent variable in the first measurement.

In the analysis of our data, we used an individual growth approach [28–31]. This method is based on hierarchical modeling, where repeated observations from a single subject represent the level-1 variables, whereas the between-subject variables are defined at level-2. According to the generally accepted practice in hierarchical modeling, we built several models in order to answer particular research questions.

One usually begins with an unconditional random intercept model to estimate the intraclass correlation coefficient (ICC). The unconditional means model was defined as follows:

$${snatch}_{ij}=\beta_{0j}+r_{ij}$$ (1)

$$\beta_{0j}=\gamma_{00}+u_{0j},$$ (2)

where *snatch*_*ij*_ represents the snatch score at time *i* for subject *j*, *β*_0*j*_ is the subject-specific intercept (the snatch score at *time* = 0), *r*_*ij*_ stands for residual error, *γ*_00_ represents the overall mean for snatch record scores, and *u*_0*j*_ is the random deviation from the overall mean. Eqs 1 and 2 represent the level-1 and level-2 models, respectively.

In the second stage of the modeling process, we defined the fixed relationship between the record snatch scores and time at the level-1 as

$${snatch}_{ij}=\beta_{0j}+\beta_{1j}{time}_{ij}+\beta_{2j}{time}_{ij}^{2}+r_{ij},$$ (3)

where *β*_1*j*_ is the subject-specific slope and *β*_2*j*_ is the subject-specific quadratic term for snatch scores over time.

The coefficients in Eq 1 can be broken down into two components at level-2:

$$\beta_{0j}=\gamma_{00}+u_{0j}$$ (4)

$$\beta_{1j}=\gamma_{10}+u_{1j}$$ (5)

$$\beta_{2j}=\gamma_{20}+u_{2j}.$$ (6)

Components *γ*_00_, *γ*_10_, and *γ*_20_ represent the mean intercept, slope, and quadratic term across all subjects, whereas *u*_0*j*_, *u*_1*j*_, and *u*_2*j*_ represent random deviations from these means for subject *j*. The random aspect of these variables indicates that they have variances and covariances, and the random intercepts, slopes, and quadratic terms may be correlated. The intercept variance was defined as *τ*_00_, the slope variance as *τ*_10_, and the quadratic term variance as *τ*_20_.

After inserting Eqs 4–6 into Eq 3, the unconditional relationship between snatch record scores and time was expressed in a compact form as

$${snatch}_{ij}=(\gamma_{00}+\gamma_{10}{time}_{ij}+\gamma_{20}{time}_{ij}^{2})+(u_{0j}+u_{1j}{time}_{ij}+u_{2j}{time}_{ij}^{2}+r_{ij}),$$ (7)

where the elements in the left bracket define the fixed part of the model, whereas the elements in the right bracket define the random part of the model.

A brief analysis of training loads (Fig 1) shows that they changed in all subjects in a similar way. It was therefore reasonable to calculate the average load for each subject and to treat it as a time-invariant covariate. Eq 4 of the final model was thus modified as follows

$$\beta_{0j}=\gamma_{00}+\gamma_{01}{\bar{load}}_{j}+u_{0j},$$ (8)

with ${\bar{load}}_{j}$ denoting subject *j*’s mean load.

Defining the final model in the above way, we assumed that mean load was treated as a predictor of the intercept. The mean loads of the subjects were also grand-mean centered in order to simplify the interpretation of the results of the computations.

We performed the statistical analysis in the R environment (R Foundation for Statistical Computing, Austria) using the *lmerTest* [32] package, which overloads the basic *lmer* function from the *lme4* package [30]. We chose the *lmerTest* package because it has several useful features, such as reporting *p*-values for *anova* and *summary* tables, testing the reduction of random-effect terms to simpler structures (*ranova* method), and performing automatic backward model selection of fixed and random parts of the linear mixed model (*step* method). The models described above were coded in R as follows:

model1=lmer(snatch∼1+(1|subject),data,REML=FALSE) (9)

$$\begin{array}{c} model2=lmer(snatch∼time+I(time^{2})+(time+I(time^{2})|subject),+\\ +data,REML=FALSE) \end{array}$$ (10)

$$\begin{array}{c} model3=lmer(snatch∼time+I(time^{2})+\bar{load}+(time+I(time^{2})|subject),+\\ +data,REML=FALSE). \end{array}$$ (11)

The terms in inner brackets denote the random parts of the models, *data* is the file in a univariate (long) format, and *REML = FALSE* means that, instead of the default restricted maximum likelihood (*REML*), maximum likelihood (*ML*) estimation was used for computations. Applying the *ML* method allows for a direct comparison between the two models nested in each other using the *anova* method [30].

The *lmerTest* package was chosen since it was assumed that subject record scores were independent and homoscedastic over time.

Significant differences in scores between time points were identified using the *gls* function (with the *corAR(1)* argument) from the *nlme* package. The use of this argument makes it possible to perform calculations for correlated measurements.

## Results

**Fig 3:**
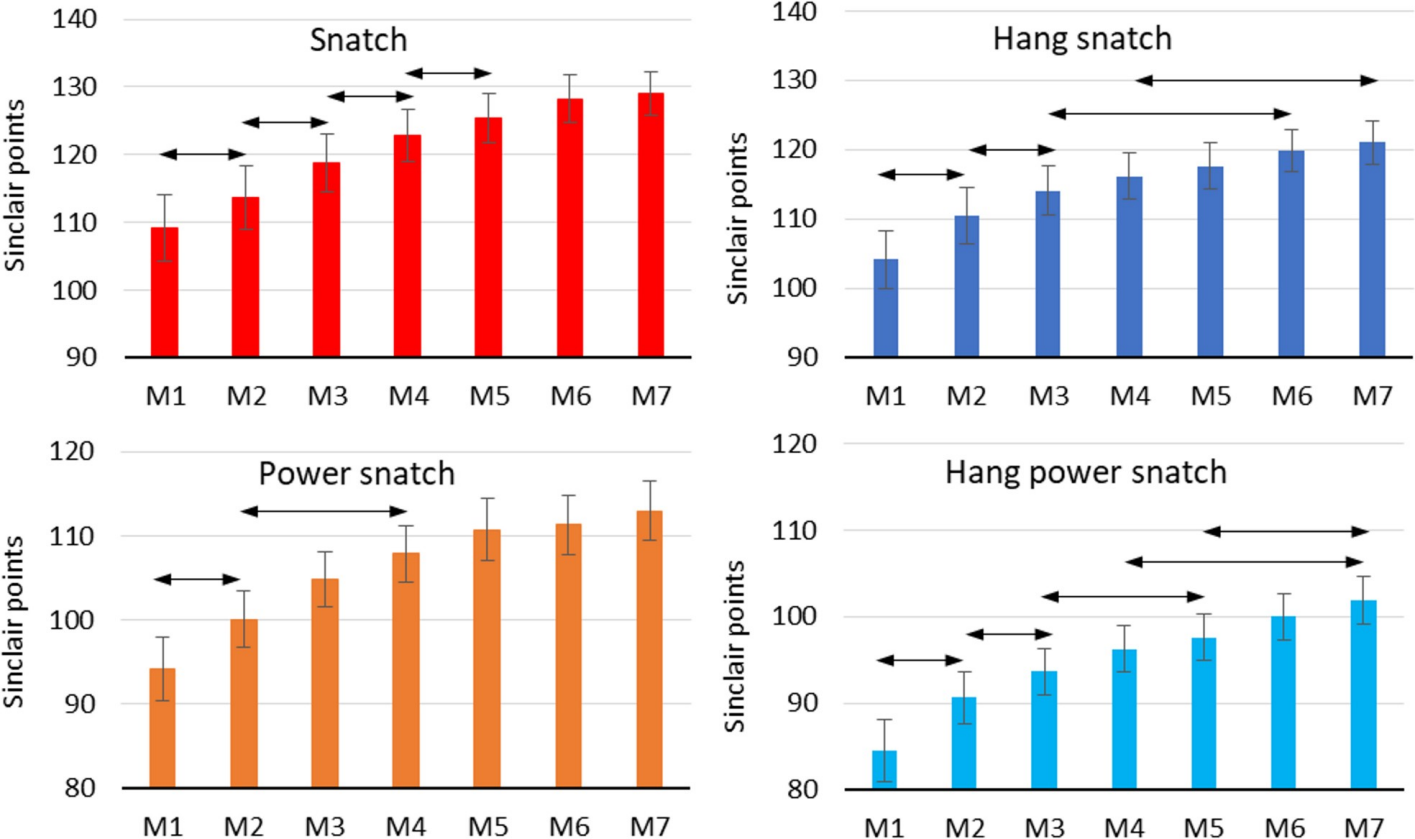
Mean record scores in the snatch and its derivatives; double arrows indicate the closest statistically significant differences.

An important element of longitudinal analyses is a preliminary examination of measurement data, which are presented in Fig 3. During the two-year training period, the lifters’ mean scores in the snatch improved by 19.78 ± 15.63 Sinclair points. As far as particular variations of the snatch are concerned, the greatest increases were found in the power snatch (18.78 ± 10.71 points), followed by the hang power snatch (17.39 ± 9.82 points) and the hang snatch (16.86 ± 11.32 points). As mentioned earlier, the subjects had at least one year’s training experience. Thus, it was reasonable to assume that the ratios of snatch derivatives to snatch record scores were approximately constant, and the overall means of these ratios were calculated. The overall mean values of the PS/S, HS/S, and HPS/S ratios were 0.88 ± 0.07, 0.95 ± 0.06, and 0.79 ± 0.07, respectively. The calculation of these ratios for the measurements (*M*_*1*_ … *M*_*7*_) confirmed the above assumption. Visual inspection of Fig 3 also reveals a non-linear time trend for the record scores in the snatch and its derivatives and a considerable decrease in these scores in the second training macrocycle.

Table 2 presents the results of statistical analysis for the models. The ICC for model 1 was 204.81/(204.81 + 90.13) = 0.69, suggesting that about 69% of the total variation in the snatch record scores was due to between-subject differences. It was evident that the relationship between snatch record scores and time could be identified using an individual growth curves approach.

**Table 2:** Estimation of model parameters.

Parameter	Model 1	Model 2	Model 3
	Coefficient (SE)		
**Fixed effects**			
Intercept (*γ*_*00*_ for *β*_*oj*_)	121.02*** (3.69)	108.85*** (4.68)	108.84*** (4.96)
Time (*γ*_*10*_ for *β*_*1j*_)		23.10*** (2.79)	23.10*** (2.81)
Time^2^ (*γ*_*20*_ for *β*_*2j*_)		−6.34*** (1.60)	−6.34*** (1.62)
$\bar{Load}$ (*γ*_*01*_ for *β*_*0j*_)			9.37* (3.96)
	Variance (SD)		
**Random effects**			
Level-1 residual (*r*_*ij*_)	204.81 (14.31)	11.98 (3.46)	11.92 (3.46)
Level-2 residuals			
Intercept (*u*_*00*_)	90.13 (9.49)	340.95 (18.47)	384.56 (19.61)
Slope (*u*_*10*_)		35.95 (5.99)	37.76 (6.15)
Quadratic term (*u*_*20*_)		4.66 (2.16)	5.48 (2.34)
**−2*LL***	867.2	707.7	703.6

* *p* < 0.05

*** *p* < 0.001

*LL*–log likelihood

The results of model 2 show that the average snatch record score at *M*_*1*_ was 108.85 Sinclair points, the average linear slope was 23.10 (Sinclair points)/quarter, and the quadratic term was −6.34 (Sinclair points)/quarter^2^. All coefficients were significant (*p* < 0.001) indicating between-subject differences in the initial snatch record scores and in the values of linear and quadratic coefficients of time trajectories. As expected, the *anova* method applied to models 1 and 2 returned a very large value of the *χ*^2^ statistic at the level of 159.5. The corresponding *p*-value of about 10^−16^ proved that model 2 fit the data considerably better. It also turned out that the value of pseudo *R*^2^, (204.81 − 11.98)/204.81 = 0.94, explained almost 94% of within-subject variability.

The results of model 3 revealed significant between-subject differences in the subjects’ intercept, slope, quadratic term (*p* < 0.001), and average load (*p* < 0.05) values. The *anova* method applied to models 2 and 3 returned a *χ*^2^ value of 4.13 (*p* < 0.05), confirming that model 3 fit the data significantly better. It is no surprise that the average trajectory for model 3 had the same coefficients as the trajectory for model 2. After subject loads were grand-mean centered, the *γ*_*01*_ coefficient did not affect the average trajectory for model 3 but influenced the intercepts of the individual trajectories.

**Fig 4:**
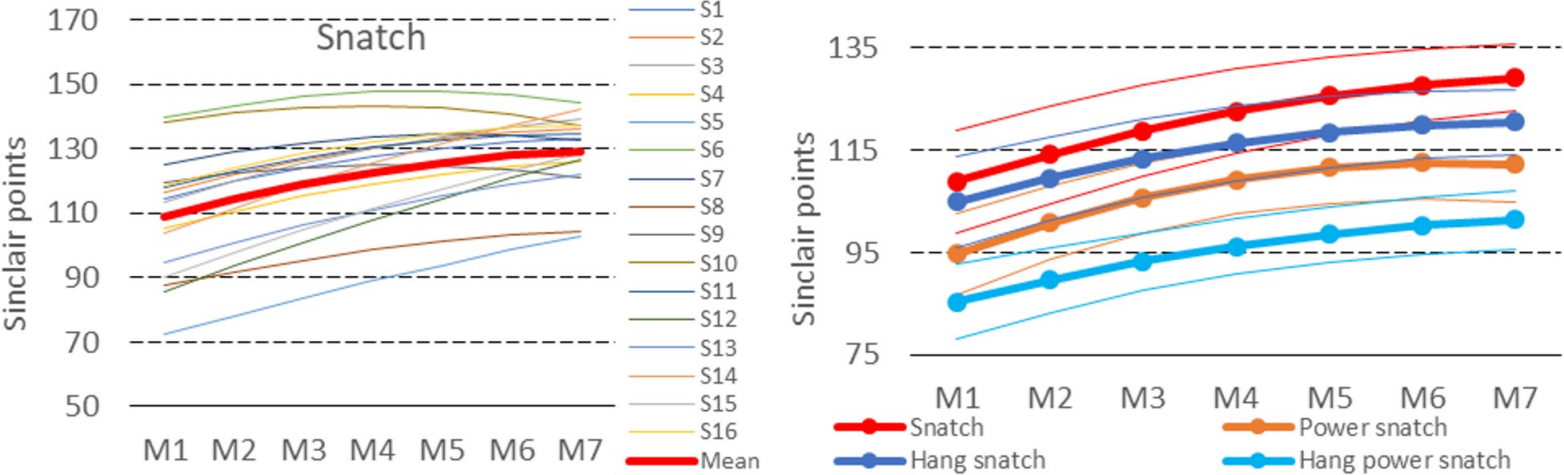
Estimated individual time trajectories in the snatch (left) and average time trajectories of the snatch and its derivatives (right) in the two-year training cycle; thin lines mark 95% confidence intervals.

Fig 4 (left) shows the time trajectories of the snatch record scores fitted with model 2. The differences between the individual trajectories and the differences between these trajectories and the average trajectory (red thick line) are clearly visible. It can also be noted that the snatch record scores of some subjects (e.g., *S*_6_, and *S*_10_) decreased in the two-year training cycle.

Derivatives of the snatch are an important component of training loads. For this reason, the trajectories of these targeted exercises were obtained using the unconditional model 2. A comparison of the average curves for the snatch and its derivatives is presented in Fig 4 (right). A similar pattern of the snatch, hang snatch, and hang power snatch curves can be easily recognized, as can a non-monotonous pattern of the power snatch curve. The values of *γ*_*20*_ coefficients for the hang snatch, the hang power snatch, and the power snatch were −6.44, −5.08, and −10.21, whereas the values of *γ*_*10*_ were 20.09, 18.32, and 27.19, respectively.

The closest significant statistical differences between the successive measurements are presented in Fig 3. The highest values of Cohen’s coefficient for correlated measurements were 1.09 for the snatch, 1.24 for the power snatch, 0.96 for the hang snatch, and 0.93 for the hang power snatch, and they occurred between the first (*M*_*1*_) and second measurement (*M*_*2*_). This indicates a large effect size in each case.

Eight trajectories were extrapolated over time without being constrained by the results at the end of the third training macrocycle. The mean relative percentage error between the actual and estimated values was 8.1 ± 3.4% for model 2, 6.7 ± 4.7% for model 3, and 9.4 ± 4.8 for ordinary least squares approximation in Excel. No statistically significant differences were found between these errors.

**Fig 5:**
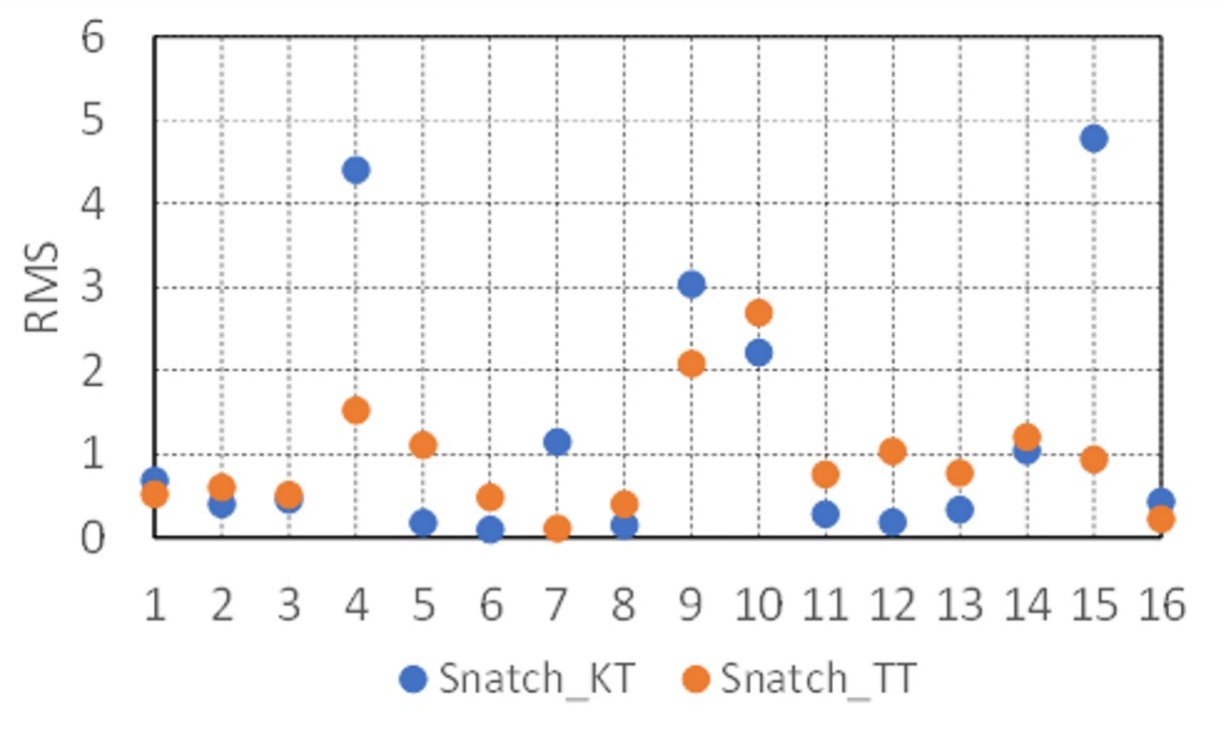
Root mean square errors between snatch record scores and isometric peak knee extensor torque (*Snatch*_*KT*) and between snatch record scores and isometric peak trunk extensor torque (*Snatch*_*TT*).

As for relationships between the snatch record scores and the isometric peak torques of the knee and trunk extensors, discrete sets of empirical data for these variables were first normalized (i.e., the mean of each variable was zero, and the standard deviation was equal to 1) in order to make it possible to compare them. The root mean square errors between the snatch record scores and isometric peak knee extensor torque and those between the snatch record scores and isometric peak trunk extensor torque were then calculated for each subject. The results of these calculations are shown in Fig 5. It is visible that lower values of root mean square error occurred between the snatch record scores and trunk extensor torque (*Snatch*_*TT*). The difference between *Snatch*_*TT* and knee extensor torque (*Snatch*_*KT*) was also statistically significant (*p* < 0.05).

## Discussion

The primary aim of the study was to identify the time trajectories of the record scores achieved in the snatch and its different variations during a two-year training cycle in young weightlifters. The calculations were made using an individual growth approach, and, in line with generally accepted practices in the field [28–30], several models were constructed. The results of the calculations showed that the method had been chosen adequately since there were statistically significant differences between individual subject trajectories, and second-degree polynomial approximation was sufficient.

The second aim of the study was to predict record scores in the snatch at the end of the following annual training macrocycle. Considering the fact that the models explained approximately 94% of within-subject variability, an absolute relative percentage error between actual and predicted values of 6.7% and 8.1% can be regarded as satisfactory. This article has described in detail the results for two models, but it is worth mentioning that several other models were tested when making the calculations. For example, one of the models used the time-varying covariate *load* instead of time as a predictor, according to the algorithm described by other authors [33–34] (−2*LL* = 730, error = 6.1%), and another model was created by combining this model with model 2 (−2*LL* = 689.3, error = 9.3%). These results and those given in Table 2 suggest that a better fit of the model to the measurement data (smaller −2*LL*) had a minor negative impact on its predictive value.

The power snatch is one of the fundamental special exercises which make it possible to improve the performance of the snatch. During the power snatch, a lifter does not need to perform a full squat when receiving the bar [6,15,16], which makes it easier to execute than the snatch. For this reason, the power snatch is often used in the training of young weightlifters [24] and non-professional weightlifters [35–36]. Higher values of coefficients *γ*_*10*_ and *γ*_*20*_ compared to those for the snatch indicate that mean increases in record scores in the power snatch occurred at a faster rate, and the trajectory of these records was more curved. Starting from measurement *M*_*6*_, there was a decrease in the values of the record scores despite a considerable increase in the training loads applied during this period. The results confirm the reports of other authors that further improvement of the results in this exercise requires an increase in lifters’ speed capacity [37] or the adequate use of other snatch variations in the training program [16].

An exercise that is often used in Olympic weightlifting training [1,15] and in crossfit training [13] is the hang snatch, in which the starting position for the barbell is at the knees, and the finishing position is the same as the one used in the snatch. An important element of this exercise is an adequate level of the flexibility angle in the ankle joint and a correct balance of the body with respect to the barbell. Thus, the hang snatch is classified as an exercise which is difficult to perform from a technical point of view. Comparable values of the *γ*_*20*_ coefficients for the snatch and the hang snatch indicate a similar curvature of the trajectory, while the somewhat higher value of the *γ*_*10*_ coefficient for the snatch evidences a higher increase in the record scores in the snatch compared to the hang snatch. The similarities between the curves also indirectly indicate that the subjects, who had minimum one-year training experience, had mastered the adequate technique of performing this exercise. This observation is additionally confirmed by the fact that the ratio of the record scores in the hang snatch to those in the snatch remained at an approximately constant level of 0.95.

Another snatch variation which is frequently implemented in strength training in different sport disciplines is the hang power snatch [19,22]. This exercise is similar to the power snatch, but the starting position of the barbell is at the knees. In specific Olympic weightlifting training, the hang power snatch is used to improve the speed of performing the second pull during the snatch. In this exercise, similarly as in the power snatch, an adequate flexibility angle in the ankle joint and a correct balance of the lifter’s body with respect to the barbell are less important. Therefore, mastering the appropriate technique of performing the hang power snatch is not difficult [37], and that is why it is used in the training of novice weightlifters. The study showed that the trajectories of the record scores in the hang power snatch were similar to those in the hang snatch and the snatch. The ratio of the record scores in the hang power snatch to those in the snatch remained at an approximately constant level of 0.79, which is an additional reason to use the hang power snatch in the training of young weightlifters and other athletes.

The largest statistically significant differences for the snatch and its derivatives were found at the beginning of the specialized weightlifting training. The statistically significant increase in record scores in the snatch was observed every 3 months until the beginning of the second training macrocycle (*M*_*5*_). This confirms the efficacy of the applied training program focused on the improvement of record scores in this lift. The significant increase in record scores in the snatch derivatives was not so regular, especially in the case of the power snatch.

An unquestionable advantage of using the snatch and its variations in the training of weightlifters and athletes practicing other disciplines is that it helps them master the habit of triple extension [1,17, 19–20]. Since at the beginning of hang snatches, the muscles of the lower limbs and trunk work in isometric conditions [38], the strength of these muscles was assessed using the isometric peak torque of these muscles. The isometric peak relative torque values of the knee extensors ranged from 4.98 ± 0.76 Nm/kg (*M*_*1*_) to 5.55 ± 1.08 Nm/kg (*M*_*7*_) and approximately corresponded with the mean values of this torque (4.86 Nm/kg) measured for the joint angle of 96° in recreational weightlifters aged 39.2 years [4]. The current values were, on the other hand, higher than those achieved by young weightlifters (4.18 Nm/kg) in the study of Jaszczuk et al. [25]. The values of trunk extensor torque changed between 6.37 ± 1.11 Nm/kg (*M*_*1*_) and 9.91 ± 2.03 Nm/kg (*M*_*7*_). Starting from *M*_*2*_ (8.29 ± 1.41 Nm/kg), they were similar to those obtained by the young weightlifters (9.19 Nm/kg) examined by Jaszczuk et al. [25].

The changes in the torque of the trunk extensors approximately corresponded with the changes in the record scores in the snatch. This would mean that the training undergone by weightlifters in the initial period of their careers leads to a synchronous increase in the strength of the trunk extensors. This observation is supported by the calculations made by Bartonietz [15], which showed that during the performance of the snatch the power generated in the hip joint is more than 3 times greater than that generated in the knee joints. Similar results were reported by Lee et al. [35], who investigated the power snatch in non-professional weightlifters.

When discussing the findings of the current study, one should emphasize the importance of at least three factors that can limit the strength of the conclusions drawn from the research. The first factor is the accuracy of determining the values of 1 RM in the snatch and its variations. The measurement resolution that was used (2.5 kg) produced errors amounting to at least 5% in the values of the record scores in the hang snatch and the hang power snatch in some of the weightlifters in *M*_*1*_ and *M*_*2*_. The second factor which made it difficult to interpret the results obtained was the use of new equipment to perform the isometric measurements of the torque of the trunk and knee extensors, for which there are no referential data for young weightlifters. Finally, we assumed subject record scores were independent and homoscedastic over time. In order to verify this assumption, we performed additional computations using the *lme* function from the *nlme* package [30], which makes it possible to check models with various forms of heteroscedasticity and autocorrelation. The computations did not show statistically significant differences (*p* < 0.082) between model 2 and its extended version containing autoregressive (*corAR1()*) and heterogeneity terms or between model 3 and its extended version (*p* < 0.074). However, low *p*-values suggest that some of the variability of the measurement data may be explained when subject record scores are non-independent and heteroscedastic over time.

## Conclusions

The results of the study revealed that the highest level of correspondence between performances in the snatch and the hang snatch as well as the snatch and the hang power snatch justifies implementing these exercises at the beginning of the careers of young weightlifters with the aim of helping them perfect the Olympic-style snatch. These exercises are also useful in other sport disciplines where learning the complicated technique of the snatch is not a priority in the training.

We believe that knowing the ratios between the record scores for the snatch and its variations may be important both for the coaches of young weightlifters and crossfit trainers. Young weightlifters make the greatest progress at the beginning of their sport career, and an adequate selection of special exercises as well as of their volume and intensity is one of the key elements of planning athletic training. Crossfit trainers, on the other hand, work with persons who have various levels of motor capacity, for whom the selection of an appropriate load is difficult, and the performance of 1 RM may be dangerous.

Tracking record scores in the snatch at regular intervals makes it possible to predict future results, and the trend in these scores is not linear in young weightlifters.

The changes in the isometric peak torque values of the trunk in the two-year training macrocycle corresponded with the changes in the record scores in the snatch, which justifies using the measurement of these torques in diagnosing the strength capacities of young weightlifters on an ongoing basis.

## Supporting information

S1 DatasetEmpirical data coded in a long format; loadBM_mean appears in the text as $\bar{load}$.(XLSX)
